# Biomarkers for Ehlers-Danlos Syndromes: There Is a Role?

**DOI:** 10.3390/ijms221810149

**Published:** 2021-09-20

**Authors:** Laura Caliogna, Viviana Guerrieri, Salvatore Annunziata, Valentina Bina, Alice Maria Brancato, Alberto Castelli, Eugenio Jannelli, Alessandro Ivone, Federico Alberto Grassi, Mario Mosconi, Gianluigi Pasta

**Affiliations:** 1 Orthopedic and Traumatology Clinic, IRCCS Policlinico San Matteo Foundation, 27100 Pavia, Italy; l.caliogna@smatteo.pv.it (L.C.); viviana.guerrieri@gmail.com (V.G.); alicemaria.brancato01@universitadipavia.it (A.M.B.); albecastell@libero.it (A.C.); eugenio.jannelli@libero.it (E.J.); alessandro.ivone87@gmail.com (A.I.); f.grassi@smatteo.pv.it (F.A.G.); mario.mosconi@unipv.it (M.M.); gianluigipasta@yahoo.it (G.P.); 2 Department of Molecular Medicine, University of Pavia, 27100 Pavia, Italy; valentina.bina01@ateneopv.it

**Keywords:** Ehlers-Danlos syndrome, biomarkers, diagnosis, follow up

## Abstract

Ehlers-Danlos syndromes (EDS) are an inherited heterogeneous group of connective tissue disorders characterized by an abnormal collagen synthesis affecting skin, ligaments, joints, blood vessels, and other organs. It is one of the oldest known causes of bruising and bleeding, and it was described first by Hippocrates in 400 BC. In the last years, multiple gene variants involved in the pathogenesis of specific EDS subtypes have been identified; moreover, new clinical diagnostic criteria have been established. New classification models have also been studied in order to differentiate overlapping conditions. Moreover, EDS shares many characteristics with other similar disorders. Although distinguishing between these seemingly identical conditions is difficult, it is essential in ensuring proper patient care. Currently, there are many genetic and molecular studies underway to clarify the etiology of some variants of EDS. However, the genetic basis of the hypermobile type of EDS (hEDS) is still unknown. In this review, we focused on the study of two of the most common forms of EDS—classic and hypermobile—by trying to identify possible biomarkers that could be of great help to confirm patients’ diagnosis and their follow up.

## 1. Introduction

Ehlers-Danlos syndromes (EDS), are a group of heritable connective tissue disorders (HCTDs) characterized by a variable degree of skin hyperextensibility, joint hypermobility, and tissue fragility, that were firstly described by Hippocrates in 400 BC [1]. The incidence of EDS is best estimated to be between 1 in 25,000 and 1 in 5000, varying greatly between the various types of EDS. For instance, the most common EDS type seems to be the hypermobile (hEDS), with an incidence ranging between 1 in 10,000 and 1 in 15000, followed by classic EDS (cEDS), which is estimated to affect 1 per 10,000 to 1 per 20,000 people [2]. Conversely, both vascular EDS (vEDS) and kyphoscoliotic EDS have a smaller incidence, ranging between 1/50,000 and 1/200,000 [3]. 

The most recent version of the EDS classification, published in 2017, recognized 13 types with 19 different causal genes mainly involved in collagen and extracellular matrix (ECM) synthesis and maintenance. Specifically, the main cause of classical and vascular EDS are defects in the genes coding for alpha chains of procollagen V and III, respectively. Whereas, the kyphoscoliotic type has been recognized to be caused by mutations either in PLOD1 or FKBP14, while the molecular background of hEDS is still unknown.

Classical (cEDS) and hypermobile (hEDS) are the most frequent types [4].

Currently, the diagnosis of EDS classical type is established by clinical examination and family history, but clinical recognition of EDS is not always linear. Frequently, molecular testing is necessary for a definite diagnosis, especially in patients with an uncertain phenotype.

Due to diagnostic difficulties, in this review we evaluated the potential diagnostic use of some biomarkers associated with the clinical picture of EDS diseases, which could be used in the future for a more accurate diagnosis, prognosis, and follow up of EDS patients.

## 2. Materials and Methods

A review of the literature was performed on two medical electronic databases (PubMed https://pubmed.ncbi.nlm.nih.gov and Embase https://www.embase.com, accessed on 15 July 2021). Two authors independently performed the study selection and the data extraction, and any discrepancies were discussed among the authors. The studies for this review were selected with screening through the following inclusion criteria:

- all studies were written in the English language
- all studies were in full text
- all studies were published in peer-reviewed journals

The search string in the two databases was as follows:

- Ehlers-Danlos AND molecular pathogenesis

The research has produced 121 articles in PubMed and 54 articles in Embase. The two databases share 22 articles.

We used 6 articles from the first string and 47 articles from their bibliography.

To deepen the research on biomarkers, present in literature, we have added two strings:

- Ehlers-Danlos AND joint hypermobility AND molecular markers
- Ehlers-Danlos AND diagnostic markers

The second string (full text articles in the last 10 years) produced four articles in PubMed and no articles in Embase. After reading the texts, two articles were selected and six articles were selected checking the bibliography in all articles examined.

The third string (full text in the last 5 years) produced 15 results in PubMed and four articles in Embase with only one in common. After reading the texts, one article was selected and one article was selected checking the bibliography in all articles examined.

Since it is an evolving field, we have not found many articles regarding biomarkers concerning Ehlers-Danlos Syndrome, therefore, we have searched carefully in the bibliography of the selected articles to try to find more information

## 3. Diagnosis of Classical EDS

### 3.1. Clinical Diagnosis

A classical EDS-type diagnosis is determined by considering the clinical family history and clinical examination. The diagnosis can be made by evaluating some major and minor diagnostic criteria, which are summarized in Table 1 [2]:

#### Clinical Features of cEDS

As for hEDS, even in the classical type there are different “collateral” clinical manifestations, not included in diagnostic criteria but useful in evaluating patients suspected for cEDS. In particular, there is gastrointestinal involvement, with nausea, vomiting, gastroesophageal reflux, and constipation in, respectively, about 46.5%, 30.2%, 30.2% and 37.2% [6]. Moreover, patients can show oral findings, such as poor periodontal status, with inferior labial and lingual frenulae absent in hEDS and cEDS, even without a significative meaning of this sign in the classical subtype [7]. Temporomandibular joint dysfunction is another common feature, with secondary headache as a consequence in classical EDS patients [8]. As concerned with ocular findings, cEDS patients can show macro and microstructural changes of the cornea, but without an increase in refractive errors or in keratoconus [9,10]. Even in this subgroup of patients, pain seems to play an important role in QOL, being, however, less prevalent and severe than in hEDS and related to abnormal extracellular matrix within muscle and peripheral nerve [11]. Again, cEDS patients present a cardiovascular involvement, with mitral valve prolapse and aneurysm and dissection of medium sized arteries [12,13].

### 3.2. Ultrastructural Studies with Electron Microscopy

Another diagnostic approach is based on tissue biopsy with electron microscopic analysis in order to evaluate classic abnormalities in the appearance of collagen. The morphological characterization of classical EDS biopsies through light microscopy techniques put in evidence characteristic collagen changes. The epidermis has a slight alteration in its contour. Collagen is loose and disperse with rare bundles and fibroblasts show an irregular morphology [14]. Similarly, transmission electron microscopy (TEM) showed some disorganized loose collagen fibers, with an irregular outline and various diameters in the cross sections [14]. Moreover, the high resolution of electron microscopy allows the detection of a characteristic shape of collagen fibrils in EDS affected cells, called “cauliflower” deformity [15]. However, these findings are not specific for EDS and, therefore, can’t be classified as diagnostic criteria but can support the diagnosis.

### 3.3. Biochemical Testing

One of the approaches used to diagnose the classic EDS is biochemical testing. Specifically, the tests rely on electrophoretic analysis of type I, III, and V collagens, derived from cultured fibroblasts isolated from a skin biopsy. The collagen molecules are labeled with ^14^C-proline and analyzed on sodium dodecyl sulfate polyacrylamide gel upon the electrophoretic run. Upon running, the gels are processed for fluorography dried, and exposed to an X-ray film [15,16]. Abnormal proteins of Collagen type I and III will migrate differently on the gel and are easily visible when compared with control samples. However, since fibroblasts synthesize collagen type V at low levels, the alteration in electrophoretic mobility is poorly reproducible. Thus, by evaluating the characteristics of this test, we can affirm that it is an inefficient method for routine diagnosis. However, this technique may be useful for studying other subtypes of EDS, such as the vascular, kyphoscoliotic, arthrochalasis, and the dermatosparaxis type in individuals in which clinical differential diagnosis is difficult [15,17].

### 3.4. Molecular Testing

Diagnosis largely has its basis in the identification of a collection of symptoms previously described that alert the practitioner to the possibility of Ehlers-Danlos syndrome. However, for specific subtype-level diagnosis, referral to a geneticist who can perform diagnostic genetic testing is recommended to confirm the Classical EDS type [2].

Most people with the cEDS have alterations in one of the two type V collagen genes, *col5a1* and *col5a2*. The most common mechanism is *col5a1* haploinsufficiency, due to the instability of the transcript of one allele [18]. Type V collagen is a quantitatively minor fibrillar collagen, which is present in much smaller amounts than other fibril-forming collagens, but it is widely distributed in a variety of tissues such as skin, tendon, bone, cornea, placenta, and fetal membranes [15]. After 2017, it has been established that the genetic analysis should be performed in all patients that fulfil clinical criteria for EDS or EDS suspected diagnosis as to confirm, establish, or classify the pathology [19]. Because there is a considerable allelic diversity among all forms of EDS, the sequence analysis provides a key to genotype–phenotype correlation. This gives the possibility to improve:

- management of complications, (surveillance and treatment),
- identification of other affected family members,
- pre-symptomatic diagnosis,
- transition from clinical assessment to gene-based diagnosis, allowing for personalized medicine to become the gold standard of disease management.

Currently, the genetic diagnostic approaches are becoming cheaper and affordable for the medical system [19]. Molecular investigations usually start from the analysis of genomic DNA (gDNA) and messenger RNA (mRNA), extracted from fibroblast cultures. At first, the presence of a non-functional *col5a1*-allele (“null-allele”) is evaluated by looking for polymorphic markers in the expressed region of the gDNA to determine whether both *col5a1* alleles have stable transcripts.

The *col5a1* “null-allele” test determines whether the individual is heterozygous for one of several polymorphic exonic markers in the gDNA of *col5a1.* Further, to verify whether both alleles are expressed, the cDNA of *col5a1* is tested. Then, if only one of the two alleles is expressed, it means the other is non-functional [15,17]. However, the evaluation of *col5a1* “null” allele does not identify *col5a1* gene mutations. Indeed, to investigate possible *colV* mutations, a DNA sequencing is required. Further, a linkage analysis can be offered to patients with a positive family history for classic EDS.

### 3.5. Next Generation Sequencing: New Molecular Diagnosis

Next-generation sequencing (NGS) technologies have a great potential for the diagnosis of a variety of disorders, including EDS [20]. The latest 2017 EDS classification identified 13 subtypes of EDS, in which the clinical and genetic phenotypes are often overlapping. The presence of so many pathological types make the diagnosis rather difficult, and for this reason the molecular diagnosis is even more important. New genetic techniques, such as next-generation sequencing (NGS), gave the opportunity to support clinical diagnosis and identify the genetic bases of different EDS types. Nowadays, genetic analysis is fundamental to confirm or modify the clinical diagnosis, and also to evaluate prognosis, making decisions on management and treatment strategies [21]. Also, the identification of molecular defects is extremely important to better explore the genotype-phenotype correlation [21]. Using the NGS, most diagnostic studies are carried out, in which a panel of known genes is sequenced and analyzed simultaneously. Multi-gene (NGS) panels that include the 20 EDS-related genes, and genes associated with the other overlapping connective tissue disorders, are the preferred diagnostic approach in patients with complex phenotypes or with no family history of EDS [19].

## 4. Diagnosis of Hypermobile EDS

### 4.1. Clinical Diagnosis

The Clinical Diagnosis of Hypermobile EDS Is Based on the Simultaneous Presence of Symptoms Belonging to Three Different Categories, Named Criteria (1, 2 and 3).

The clinical criteria used to diagnose hEDS are summarized in the Table 2.

#### 4.1.1. Clinical Features of hEDS

To date, the hEDS diagnosis remains only clinical as there is no reliable or appreciable genetic etiology to test for in most patients.

In addition to the characteristics already described in the diagnostic criteria, patients affected by hEDS can present a series of collateral manifestations that are often more debilitating than the symptoms affecting the various joint districts, leading to a significant impairment of QOL. These manifestations were not included in the diagnostic criteria because they are not sensitive and specific enough, but they represent a valid “differential diagnosis-tool” between hEDS and other pathological conditions that present a clinical overlap.

Among these features there are: pelvic organ prolapse (POP), pregnancy, and menstrual cycle complications (for example infertility, which were found in 10% of general population but in 44% of EDS patients, with hEDS being the most involved) [23], as well as autonomic dysfunction with cardiovascular, gastrointestinal, and genito-urinary manifestations, such as tachycardia, postural orthostatic tachycardia syndrome (POTS), vasovagal syncope or neurally mediated hypotension (NMH), orthostatic hypotension (OH) [24,25], abdominal pain, nausea, reflux symptoms, vomiting, constipation, diarrhea [26] and impaired bladder function, sleep disturbance, with insomnia and poor sleep quality (common in children and adults) [27,28,29], fatigue, anxiety, depression [2] and pain.

Concerning the pain, Sacheti et al. reported that 100% of 28 hED studied patients suffered from pain, with a mean score on the NRS of 8 out of 10 for all types of EDS [30], showing the importance of this symptom. Similar values were found by Voermans et al., with 90% of all 273 EDS studied patients and 93% of them reported joint hypermobility, who even showed the highest scores on severity of pain [11]. Pain seems to be inversely correlated to generalized joint hypermobility; in fact, approximately 30% of children with diagnosed hEDS reported arthralgias, back pain, and myalgias, while this rate became more than 80% when considering patients over the age of 40 years, who often show a “negative” Beighton score but increased pain [31]. Pain may be localized or widespread and may be acute or chronic. It is frequently localized in the neck, shoulders, hips, forearms, and legs. At the beginning, pain seems to be limited to a few joints and/or muscles and shows a migratory pattern, but then it becomes more persistent with a generalized distribution. Patients describe pain as a burning sensation, peripheral paresthesia, generalized hyperalgesia, allodynia, and a hypersensitivity to different stimuli [32,33,34,35]. Even gastrointestinal, genito-urinary, and pelvic areas are affected by pain [30,35,36,37,38,39,40,41,42].

#### 4.1.2. Pain in cEDS and hEDS

The genesis of the pain in these patients has not yet been fully understood. It seems to be related to nociceptive and neuropathic pain, with a component of pain sensitization. In particular, nociceptive pain is found during early stages of acute and localized pain and it is related to affected ligaments and tendons, joints muscles, and connective tissue. Ligamentous and tendinous damage, which derives from joint instability, and the presence of microtrauma on joint surface, which in turns lead to adaptations and overload in other areas, contribute to nociceptive pain [43,44]. On the other hand, the neuropathic component for HEDS pain is due to compressions and axonal neuropathies in different EDS subtypes [45]. In fact, this type of pain can be explained by nerve (sub)luxations that are found to be more frequent in the upper limb area and can explain neuropathies such as paresthesia [32]. In addition, a decreased intraepidermal nerve fiber density was demonstrated in skin biopsies in these patients, which helps to explain nerve neuropathies [46,47]. The last contribution to chronic pain seems to derive from central sensitization, which was found in many patients, suggesting a generalized hyperalgesia in patients suffering from these conditions [34,35]. This was confirmed by a research group who showed that HEDS/JHS has increased the wind up to repeated stimuli and decreased exercise-induced analgesia. Central sensitization could be considered as a consequence of the continuous stimulation of peripheral nociceptors by mediators released by the aberrant Extracellular Matrix (ECM) [48].

In conclusion, pain seems to recognize different causes in different phases of the syndrome: initially it could be due to joint hypermobility complications, but these persistent nociceptive inputs may cause central sensitization in the dorsal horn neurons and abnormalities of the endogenous pain inhibitory control [49].

### 4.2. Genetic Findings in hEDS

Many genes were studied to identify hEDS’s causal factors, but little was found, showing the heterogeneous origin of these conditions. Furthermore, these variants have only been found in a small percentage of hEDS patients, leaving most cases without a genetic diagnosis.

Among these, in a single family, a point mutation (with glycine 637 to serine substitution) in *col3a1* was found, such as what has been observed in vEDS patients, only without the vascular phenotype. It led to a reduced and altered collagen organization and segregated in an autosomal dominant manner [50].

Again, into a multigenerational Belgian family a heterozygous missense mutation in a chromosomal locus (8p22–8p21.1) of the *lzts1* gene was confirmed to be present in all hEDS patients and in none of the unaffected, but its involvement in connective tissue biology is still not clarified and additional research needed to be done in this way [51]. The screening of hEDS patients revealed three additional *lzts1* variants, a gene encoding for leucine zipper tumor suppressor 1, whose pathogenic variants are associated with several types of cancers.

Another gene found to be mutated in hEDS is *tnxb*, a gene encoding for Tenascin X, an extracellular matrix protein involved in maintaining the distance between collagen fibrils by forming bridges through direct interactions with collagen fibrils [52,53,54,55]. While truncating mutations and deletions in *tnxb*, resulting in Tenascin X deficiency [56,57], were found in patients with major and minor diagnostic criteria for cEDS type [56], *tnxb* haploinsufficiency has been found in patients with an autosomal dominant form of hEDS who did not show easy bruising and skin hyperextensibility, like in *tnx* deficiency [58]. Zweers et al. found that reduced tenascin-X serum levels were only present in 5–10% of patients diagnosed with Benign Joint Hypermobility Syndrome, or hEDS [59]. T*nxb* deletion can even be caused by a contiguous deletion syndrome (CAH-X) [60], in which patients have a 21-hydroxylase deficiency causing congenital adrenal hyperplasia and hEDS in which *tnxb* exons are replaced by *tnxa*, giving birth to *tnxa/tnxb* chimeras [60,61,62].

Other gene mutations concern *tpsab1*, a gene encoding for alpha-tryptase, whose high levels were found in patients with symptoms easily found in hEDS patients (such as connective tissue abnormalities, autonomic dysfunction, gastrointestinal disorders, allergic, and cutaneous symptoms), although not all patients with *tpsab1* mutations met diagnostic criteria for hEDS and elevated alpha-tryptase basal serum levels are quite common in the general population [63].

### 4.3. Ultrastructural Studies with Electron Microscopy

The microscopic anomalies concerning the extracellular matrix, in particular the collagen fibers and the elastic fibers, in patients with hEDS do not seem, to date, to be the basis for the diagnosis of these pathological conditions. In patients with hEDS and low values at the Beighton score evaluation, anomalies of the structure of the collagen fibers were found—i.e., a reduced thickness and a fibrillar disorganization and a “flower-like” appearance of the fibrils, without there being a correlation between the anomalies observed and the degree of hypermobility [64,65,66,67,68]. The anomalies, in addition to the structure, concern the quantity of collagen fibers, in particular the ratio between collagen type I/collagen type IIII. Even elastic fibers also show non-dirimental alterations. An electron microscope (TEM) study recently conducted by Angwin and colleagues showed no specific correlation between collagen abnormalities and the different EDS subtypes, other than the presence of collagen flowers in many cEDS patients. In particular, many of the patients suffering from hEDS presented normal biopsies on TEM despite the presence of anomalies justifiable as age-related variants [69].

### 4.4. Gene Expression Analysis

As previously stated, although today the genes underlying hEDS have not yet been identified with certainty, alterations have been found in the gene expression of molecules involved in numerous cellular processes, such as cell adhesion (eg, *cldn11*, *dsp*, *flg*, *itga4*, *itga2*, *cdh10*, *cdh2*, *pcdh9*, *pcdhb16*, *pcdhb8*), the signal transduction system (eg, *aqp9, chrm2*, *clic2*, *kcnq5*, *opcml*, *prlr*, *slco2a1*, *npr3*), redox homeostasis (eg, *adh1b*, *adh1c*, *akr1c3*, *gstm5*), and numerous inflammatory processes [70] which, as mentioned above, allow us to explain and understand the origin of most of the collateral manifestations of EDS.

Chiarelli and colleagues analyzed proteome profiling of hEDS patients’ dermal myofibroblast and they found 183 DEPs. These proteins were involved in cellular metabolism and redox homeostasis (19%), cytoskeleton organization (16%), translation (14%), protein modification (8%), membrane trafficking (8%), calcium binding (7%), transport (7%) and chaperon (4%), and adaptor (4%) and nucleic acid binding (3%) functions [71].

Since hypermobility is one of the diagnostic criteria of hEDS, many authors have focused their attention on this pathological condition. Tuna and colleagues studied which gene expressions and serum levels are altered in GJH, with the aim of highlighting the positive correlation with the Beighton score, trying to identify altered gene and molecular expressions that could easily be found even in patients with EDS. The results are reported in the table below (Table 3) [72], with the highlighted parameters showing a significant positive correlation with the Beighton Score, at the Pearson correlation analysis.

### 4.5. MicroRNAs Profiling

There are many miRNAs whose expression is altered in hEDS, which can contribute to decreased mRNA levels of SFRP2 and consequently an aberrant signaling of the Wnt/β-catenin axis, playing a role in hEDS pathogenesis. Among these miR-378-3p, a modulator of fibroblast-myofibroblast transition, associated with inflammation and fibrosis through NF-kβ and TNFⲁ pathways [73,74]; miRNA-224, associated with the activation of Wnt/β-catenin signaling through the inhibition of the expression of glycogen synthase kinase 3β and SFRP2 [75]; decreased expression of miRNA-23, involved in Wnt pathway regulation by inhibiting the expression of FDZ5 and FDZ7 receptors [76].

## 5. Future Perspective in EDS Diagnosis: Possible Biomarkers

As previously mentioned, Ehlers-Danlos syndromes (EDS) are a group of heritable connective tissue disorders. EDS are difficult to identify because they present many features in common with other Heritable Connective tissue disorders (HCTDs), such as Marfan syndrome and some types of skeletal dysplasia and cutis laxa. For these reasons, it is necessary to use molecular tests that can be essential to identify patients with an uncertain phenotype to clarify and confirm the EDS diagnosis. EDS have clinical and genetic heterogeneity, but the subjective interpretation of some semiquantitative clinical signs, such as skin hyperextensibility, skin texture, JHM, tissue fragility, and bruising, led to diagnostic ambiguity and confusion regarding the type of EDS [77]. EDS patients are often affected by joint hypermobility syndrome (JHS) and joint inflammatory diseases (Inflammatory Arthritis, Rheumatoid Arthritis). Then, to clarify the clinical picture, confirm further the diagnosis, and follow the evolution of the disease, biomarkers associated with clinical pictures of these diseases could be used.

### 5.1. Serum Complement Proteins

The complement system plays specific roles as a part of innate immunity. It is not only involved in host defense recognition and the elimination of potentially microbial pathogens, but also in different forms of acute and chronic inflammatory diseases, such as sepsis and rheumatic disease. The regulation of the complement system is a useful strategy to control inflammatory diseases [78,79]. In JHS, interventions have been limited to symptomatic treatments, and biomarkers for diagnosis and therapy have not yet been identified. In a recent study, it was identified potential serum biomarkers for JHS. The researchers have examined six different proteins (some of the complement) with differential levels in serum from patients with JHS and from control individuals [79]. The six proteins involved in the study were:

- C1R subcomponent (C1R): component of C1 complement protein. It is the serine protease responsible for intrinsic activation of the C1 complement protein.
- Vitronectin (VTN): is a plasma multifunctional glycoprotein that is implicated in cell migration, blood coagulation, fibrinogenesis, the inflammatory process, and membrane attack complex (MAC) formation. VTN also acts as an inhibitor of the cytolytic reactions of MAC [79].
- Complement component C9 (C9): component of MAC. VTN binds directly the C5b-7 complex and C9, on distinct binding sites.
- C4B-binding protein alpha chain (C4BPA): C4BP is a potent circulating soluble inhibitor of the classical and lectin pathways of the complement system. C4BPA binds to C4BP, and this interaction inhibits the complement activation pathway by reducing the formation and stability of C4BC2b (C3 convertase) [79].
- Apolipoprotein B-100 (APOB): is a ligand for the low-density lipoprotein (LDL) receptor that participates in cholesterol transport to peripheral tissues and its accumulation in the arterial wall [79].
- Transthyretin (TTR): is a carrier for thyroxine and retinol-binding protein (RBP) [79].

The Inflammation associated with joint instability, degenerative joint disease, and chronic pain is common in patients with JHS and EDS. In these patients, the analysis of C1R, VTN, C9, and C4BPA proteins in their serum showed higher levels of protein as control individuals [79]. It has been reported that C1R is synthesized and secreted in cell cultures of synovia from patients with rheumatoid arthritis, indicating the involvement of C1R in the inflammatory process in rheumatoid arthritis [80]. The complement protein C9 deposition was noted in the synovial vasculature of patients with acute arthritis and rheumatoid arthritis [79]. The six proteins identified in the study also include APOB and TTR. The plasma concentration of APOB is known to be a good marker of cardiovascular risk [81], and its increased levels have been observed in patients with osteoarthritis [82]. Wilson [83] has reported that exome analysis of a patient with EDS classical type (type I) revealed three heterozygous mutations in TTR, fibrillin 1 (FBN1), and voltage-gated calcium channel subunit alpha Cav2.1 (CACNA1A) genes.

Moreover, the C5a complement protein was investigated. C5a is an active fragment produced during complement activation, is a strong chemotactic factor, and plays important roles in various inflammatory processes. In patients with rheumatoid arthritis, the increase of C5a levels in serum or joint fluid was correlated with more severe inflammation [78]. C3a, another protein complement, is present at elevated levels in synovial fluid in rheumatoid arthritis. It is interesting to note that C5a and C3a do not increase in the synovium of patients with osteoarthritis [78]. Moreover, C5a and C3a play roles in pain; they activate and sensitize cutaneous nociceptors. This illustrates that C5a and C3a are involved in pain [84]. Considering that, the Ehlers-Danlos patients often suffer for JHS, and the evaluation of expression levels of some complement factors may contribute confirm the diagnosis and assess the level of pain related to the severity of the disease.

As we have seen earlier, proteomic analysis in JHS serum patients showed increased levels of different proteins of the complement system such as C9, C1R, and vitronectin [79]. The analysis of Hypermobile EDS fibroblast shows an increased expression of another complement factor: complement factor D (CFD). CFD is a serine protease and is a component of the alternative complement pathway that has a role in the inflammatory process. CFD is involved in pathophysiological mechanisms of osteoarthritis and has been considered as a potential predictive biomarker of joint pain in patients with hip and knee osteoarthritis [4,85].

In view of some data present in the literature, other complement factors could prove useful biomarkers to confirm the diagnosis and follow up of the patient with Ehlers-Danlos.

### 5.2. Acquaporins 9 (AQP9) and Interleukin 6

The transcriptome analysis of skin fibroblast of hEDS patients showed increased expression aquaporins 9 (AQP9). AQP9 is a member of aquaporins that enhances skin barrier function and antimicrobial defenses. A microarray study on peripheral blood mononuclear cells of patients with irritable bowel syndrome, psoriasis, and rheumatoid arthritis, identified AQP9 as a novel marker of chronic inflammation typical of these diseases [85]. Synovial tissues and fibroblast-like synoviocytes from osteoarthritis and rheumatoid arthritis patients were detected to have a high expression of this protein. This suggests how AQP9 may have a role in the pathogenesis of inflammatory synovitis [4].

A functional analysis of JHS/hEDS fibroblast patient’s show how Interleukin 6 (il6) gene is down regulated [85]. IL6 is a cytokine implicated in immune and inflammatory responses and its dosage could be an additional useful tool for studying the disease and its follow up.

### 5.3. Prolactin

Literature data show how elevated serum prolactin (PRL) levels were associated with a variety of pain conditions (migraine, burning, rheumatoid arthritis, and osteoarthritis) and can modulate the activity of nociceptors, playing an important role in pain responses and inflammation. As EDS patients often suffer widely of chronic pain related to JHS, it is reasonable to suppose that the PRL might be involved in that [85]. Given the link between this protein and chronic pain, which unfortunately affects a large part of Ehlers-Danlos patients, it would be interesting to deepen the studies to verify the possibility of using its dosage as a new biomarker.

### 5.4. Selenium Binding Protein

Selenium-binding protein-1 (SELENBP1) is involved in selenium transport, an essential nutrient that displays neuroprotective and antioxidant activities in preventing certain neurologic diseases, such as schizophrenia and bipolar disorder. In literature there are data that show how the up-regulated expression of SELENBP1 in both blood and brain of schizophrenic patients results in a strong candidate biomarker for schizophrenia [86]. Considering that EDS patients often suffer for psychiatric disorders, such as psychosocial impairment, reactive depression, mood and obsessive-compulsive [87,88], it would be interesting to investigate the possibility of measuring SELENBP1 presence in EDS blood patients as a biomarkers to confirm the diagnosis.

### 5.5. miRNA

Whole transcriptome analysis aims to analyze both coding and non-coding RNA and quantifying gene expression heterogeneity in cells and tissues. This analysis is important because it gives us the opportunity to understand the genetic interaction networks to clarify cellular functions, growth/development, and biological systems [89]. miRNAs are small non-coding RNA molecules ranging from 20–25 nucleotides in length that act mainly as negative regulators of gene expression by promoting the degradation of target mRNAs or repressing their translation [90,91]. Several pathological conditions, such as cancer, musculoskeletal disorders, painful peripheral neuropathies, and fibromyalgia have been identified as the aberrant miRNA expression.

Recently, Chiarelli et al. [4] offers a wide overview on molecular mechanisms likely involved in the classical, vascular, and hypermobile Ehlers-Danlos Syndrome, which could direct future studies to possible therapeutic strategies. The authors review their previous transcriptome and protein studies on dermal fibroblasts from cEDS, vEDS. and hEDS patients, providing information on their molecular mechanisms. These cells, despite sharing a common ECM remodeling, show differences in the underlying pathomechanisms [4]. The pathological ECM turnover in cEDS and vEDS dermal fibroblasts, is directly caused by molecular defect causing abnormal expression of collagen V and collagen III, which, consequently perturbs the physiological processes for collagen processing itself and the maintenance of cell homeostasis [4]. Since we are not yet aware of hEDS disease etiology, we must assume that the abnormal ECM organization present in hEDS cells may be a functional consequence of excessive remodeling due to increased levels of ECM-degrading enzymes and concomitant acquisition of a pro-inflammatory myofibroblast-like phenotype. The study of the transcriptome for the first time has allowed understanding different biological disease aspects. Furthermore, the understanding of transcriptional changes of genes and miRNA involved in molecular pathways related to pain and inflammatory response, might explain the biological pathways involved in chronic and musculoskeletal pain affecting hEDS/HSD patients [4].

The application of proteomic approaches is a new tool to identify the complex protein network and pathways involved in EDS pathogenesis. Further investigations on proteomic profiling of EDS patients could allow us to identify potential biomarkers or potential bioactive molecules involved in the disease that may be supportive to the clinical diagnosis and therapy of this disorder [4,85].

### 5.6. Urinary Biomarkers

It has been observed a new form of Ehlers-Danlos syndrome (EDS, Musculo-contractual type 1), which is caused by mutations in the carbohydrate sulfotransferase 14 gene (chst14) encoding CHST14/dermatan 4-Osulfotransferase-1 (D4ST1). The chst14 gene is responsible for the biosynthesis of Dermatan sulfate (DS), that plays important roles in many biological activities such as cell signaling, tissue morphogenesis, and interactions with various extracellular matrix proteins such as collagen.

Recently, Mizumoto et al. [92] investigated the presence and the quantification of DS in urine of patients with homo- or compound heterozygous mutations in chst14. DS was absent in the urine and these results suggest that their absence is a non-invasive biomarker for the diagnosis of this EDS type.

Another rare type of EDS is the kyphoscoliotic type of Ehlers-Danlos syndrome (EDS VIA), which is characterized by a deficiency of collagen lysyl-hydroxylase 1 due to mutations in plod1. Biochemically, this results in the underhydroxylation of collagen lysyl residues and, hence, an abnormal pattern of lysyl-pyridinoline (LP) and hydroxylysyl-pyridinoline (HP) crosslinks excreted in the urine. Rohrbach [93] shows how the urinary pyridinolines analysis is a fast and non-invasive diagnostic test for this type of Ehlers-Danlos syndrome.

Although Classical and Hypermobile Ehlers-Danlos syndrome—what this review has focused on—have different genetic characteristics compared to the two described above, we can hypothesize that performing further studies on urine analysis of cEDS and hEDS patients could be interesting to check if there were any characteristics that could allow the discovery of non-invasive diagnostic biomarkers.

## 6. Discussion

Ehlers Danlos-syndrome is a group of hereditary connective tissue disorders, which manifests clinically with soft and hyperextensible skin, abnormal wound healing, easy bruising and joint hypermobility. The fragility of soft tissues, fragility of vessels and hollow organs, and the involvement of the musculoskeletal system are additional clinical characteristics that differ among EDS subtypes [19]. All these clinical features lead to a situation of severe disability and poor quality of life for patients and their families. The disease has mainly been diagnosed by evaluating clinical features, and the molecular analysis is necessary to confirm and identify the type of EDS. In 2017, the International EDS Consortium published a new International Classification System [2] in considering that there is a heterogeneity and overlap between EDS subtype and clinical patients features, and that there are no specific medical or genetic therapies for any type of EDS patients. The new classification describes clinical criteria for each subtype and also presents guidelines for genetic and molecular diagnostic confirmation for all EDS subtypes, except for the hypermobile type [2]. Learning about the EDS type is important for patient therapy and to guide management and counseling. Since the pathology is a connective disorder, the affected tissues and organs are widespread throughout the body, therefore this can make EDS diagnosis and management difficult.

Management, consisting of treatment, surveillance, and the prevention of complications, requires a multidisciplinary approach involving multiple subspecialties in order to ensure optimum care [19]. Each clinician who takes care of a patient with Ehlers-Danlos syndrome should consider the multitude of complications of the disease and the potential preventative measures.

To date, there are no therapy, treatment, and management protocols for EDS patients. Clinicians should use a multidisciplinary approach that focuses on the prevention of disease progression and subsequent complications. The clinicians attempt to prevent or slow the progression of the disease, they should suggest to the patients to prefer specific lifestyles and do not leave any physical or medical treatment. For the EDS patient, it is important that they receive psychosocial support; in fact, they must be helped to understand that there is no recovery for their disease [15]. Many years have passed since in the early twentieth-century when Edvard Ehlers and Henri-Alexandre Danlos described patients with joint hypermobility, excessive skin extensibility, easy bruising, and abnormal scar formation after injury, but the search field is still open to clarify many aspects of this heterogeneous group of connective tissue disorders.

In this review, we tried to identify potential biomarkers by reading and looking for the few data that are currently in the literature. The EDS diagnosis is mainly clinical, and the molecular tests have been used to confirm or to establish the classification. Unfortunately, we have found few jobs that have studied the possible biomarkers for diagnosis and follow up and consequently EDS etiology research is a field still unexplored. In literature, we found some complement factors that increase in the presence of JHS (pathology that often afflicts EDS) and how their dosage may be useful to confirm the diagnosis. Their increase or decrease may be a possible marker of worsening or improvement of the disease, showing a new possible biomarker for patient follow up.

The analysis of the hEDS patients’ fibroblasts showed that there is a greater expression of AQP9 gene and down regulation of IL6 gene. AQP9 and IL6 are involved in the inflammatory process, since EDS patients frequently show chronic disease, AQP9 and IL6 could be use as possible diagnostic biomarkers.

In addition, PRL may have a role in inflammation and their serum levels could suggest a clinical situation of chronic inflammation that is frequent in EDS patients. Scientific data show the up-regulation of SELENBP1 in schizophrenic patients. Considering that EDS patients suffer from psychiatric disorders, it would be interesting to dose SELENBP1 in serum EDS patients.

Recently, all transcriptome analysis offers a wide overview on molecular mechanisms likely involved in EDS syndrome. The understanding of genes and miRNA changes might explain biological mechanisms involved in EDS patients’ clinical picture. As explained above, recent studies about EDS musculocontractural type 1 and kyphoscoliotic type (EDS VIA) showed an abnormal protein urinary pattern. It would be interesting to perform laboratory and clinical trials studies on urine analysis of EDS patients to check if there is a specific pattern.

## 7. Conclusions

Further laboratory studies and clinical trials would be carried out in order to obtain information about the use of these potential biomarkers, both in the diagnostic phase and to evaluate the evolution of a pathological condition very often not recognized in useful times. The diagnosis of this pathology is made even more difficult by the extreme variety of symptoms reported or manifested by ED patients. For this reason, we believe that knowledge of such a varied and often little-known syndrome is the only way to ensure adequate treatment for affected patients, without prejudice, to the need to investigate new potential biomarkers, albeit not specific for this pathological condition.

## Figures and Tables

**Table 1:** Clinical diagnosis of Classical EDS.

The Clinical Diagnosis of cEDS Requires the Simultaneous Presence * of: Major Criterion (1) + Major Criterion (2) or Major Criterion (1) + At Least 3 Minor Criteria	
Major Criteria	Minor Criteria
Skin hyperextensibility ^1^ and atrophicscarring ^2^ Generalized joint hypermobility (GJH) ^3^	Easy bruising ^4^ Soft, doughy skin ^5^ Skin fragility (or traumatic splitting) Molluscoid pseudotumors ^6^ Subcutaneous spheroids ^7^ Hernia (or history thereof) Epicanthal folds ^8^ Complications of joint hypermobility (e.g., sprains, luxation/subluxation, pain, flexible flatfoot) Family history of a first degree relative who meets clinical criteria

* Confirmatory molecular testing is mandatory to reach a final diagnosis; ^1^ Skin extensibility should be measured by pinching and lifting the cutaneous and subcutaneous layers of the skin on the volar surface at the middle of the non-dominant forearm [5]. Skin is hyperextensible if it can be stretched over a standardized cut-off in three of the following areas: 1.5 cm for the distal part of the forearms and the dorsum of the hands; 3 cm for neck, elbow, and knees. ^2^ Abnormal scarring can range in severity. Most patients have extensive atrophic scars at a number of sites. These can sometimes be haemosiderotic. A minority of patients are more mildly affected. ^3^ GJH is evaluated according to the Beighton score; a Beighton score of ≥ 5 is considered positive for the presence of GJH. Since laxity decreases with age, patients with a Beighton score of < 5/9 may be considered positive based on their historical observations, by using “five-point questionnaire” (5PQ). ^4^ Easy bruising can occur anywhere on the body, including unusual sites. The pretibial area often remains stained with hemosiderin from previous bruises. ^5^ Subjective abnormality of the skin texture is appreciable by touching the skin. ^6^ Molluscoid pseudotumors are fleshy lesions associated with scars and found over pressure points (e.g., elbow, fingers). **^7^** Subcutaneous spheroids are small spherical hard bodies, frequently mobile, and palpable on the forearms and shins. Spheroids may be calcified and detectable radiologically. ^8^ Epicanthal folds are often seen in childhood but may also be seen in adults.

**Table 2:** Clinical Diagnosis of Hypermobile EDS.

The Clinical Diagnosis of hEDS Requires the Simultaneous Presence of 3 Criteria (1, 2 and 3)	
Criterion 1	Generalized joint hypermobility (GJH), defined by Beighton Score (BS). GJH is diagnosed with ➣ BS ≥ 6 for pre-pubertal children and adolescents ➣ BS ≥ 5 for pubertal men and women up to the age of 50 ➣ BS ≥ 4 for those >50 years of age
Criterion 2 (Two or more among the features A–C must be present)	**SIGN A** (five or more of the following manifestations should be present): Unusually soft or velvety skin Mild skin hyperextensibility ^1^ Unexplained striae ^2^ Bilateral piezogenic papules of the heel ^3^ Recurrent or multiple abdominal hernia(s), such as umbilical, inguinal or crural Atrophic scarring involving at least two sites and without the formation of truly papyraceous and/or hemosiderotic scars ^4^ Pelvic floor, rectal, and/or uterine prolapse in children, men or nulliparous women Dental crowding and high or narrow palate ^5^ Arachnodactyly ^6^ Arm span-to-height ≥ 1.05 Mitral valve prolapse mild or greater based on strict echocardiographic criteria Aortic root dilatation with Z-score > +2 **SIGN B:** Positive family history, with one or more first degree relatives independently meeting the diagnostic criteria for hEDS **SIGN C** (at least one): Musculoskeletal pain in two or more limbs, recurring daily for at least 3 months Chronic, widespread pain for ≥3 months Recurrent joint dislocations or frank joint instability, in the absence of trauma
Criterion 3 (All must be met)	1. Absence of unusual skin fragility, which should prompt consideration of other types of EDS 2. Exclusion of other heritable and acquired connective tissue disorders (HCTDs) (based on history, physical exam, and/or molecular genetic testing), including autoimmune and rheumatologic conditions 3. Exclusion of alternative diagnoses that include joint hypermobility by means of hypotonia and/or connective tissue laxity

^1^ Mild skin hyperextensibility, assessed at a site lacking excess or loose skin and without evidence of prior trauma by gently pulling until resistance is met. An ideal location is the volar surface of the non-dominant forearm, where the upper limit of normal extensibility is 1.5 cm [5]. Extensor surfaces of joints have excess skin and should not be used. More significant extensibility (e.g., >2.0 cm) should prompt consideration of other EDS types, especially in combination with other cutaneous features, such as papyraceous scars, molluscoid pseudotumors and/or subcutaneous spheroids. ^2^ Unexplained (because there is not a history of significant gain or loss of body weight) striae distensae or rubrae at the back, groins, thighs, breast, and/or abdomen in adolescents, men, or prepubertal females ^3^ Bilateral piezogenic papules (herniations of subcutaneous heel fat visible upon standing) of the heel; these must be present bilaterally to be considered positive. These are rarely found in children but can be easily found in adults with history of prolonged standing (occupational) [22], marathon runners or weightlifters. ^4^ Atrophic scarring involving at least two sites and without the formation of papyraceous and/or hemosideric scars. Atrophic scarring is defined as scars from linear traumatic lacerations or single surgery that are unusually shallow and/or wider than the original wound. Atrophic scars deriving from multiple skin incision, wound infection or inflammatory conditions are to be considered negative ^5^ Dental crowding (including a history of crowding corrected by orthodontia) and high or narrow palate. Both conditions must be positive to count toward this feature. ^6^ Arachnodactyly, defined as either bilateral positive wrist sign (Steinberg sign) or bilateral positive thumb sign (Walker sign).

**Table 3:** Impaired gene expression in women with GJH.

Impaired Gene Expression in Women with GJH	
Higher Levels	Lower Levels
**TNXB**	COL1A1
**B3GALT6**	COL1A2
B4GALT7	DSE
**SLC39A13**	FKBP14
**Zn**	Li
**Sr**	

Bold to stress which biomakers shows singinficant correlation.

## Data Availability

https://pubmed.ncbi.nlm.nih.gov (accessed on 15 July 2021); https://www.embase.com (accessed on 15 July 2021).
